# The promotion of nephropathy by *Porphyromonas gingivalis* lipopolysaccharide via toll-like receptors

**DOI:** 10.1186/s13098-017-0271-8

**Published:** 2017-09-22

**Authors:** Koichiro Kajiwara, Shunsuke Takata, Thao T. To, Kenyo Takara, Yuji Hatakeyama, Sachio Tamaoki, Richard Peters Darveau, Hiroyuki Ishikawa, Yoshihiko Sawa

**Affiliations:** 1 Department of Oral Growth & Development, Fukuoka Dental College, 2-15-1 Tamura, Sawara-ku, Fukuoka, 814-0193 Japan; 2 Department of Periodontics, University of Washington School of Dentistry, 1959 NE Pacific St, Seattle, WA 98195 USA; 3 Department of Morphological Biology, Fukuoka Dental College, 2-15-1 Tamura, Sawara-ku, Fukuoka, 814-0193 Japan; 4 Department of Periodontics & Microbiology, University of Washington School of Dentistry, 1959 NE Pacific St, Seattle, WA 98195 USA; 5 Fukuoka Dental College, 2-15-1 Tamura, Sawara-ku, Fukuoka, 814-0193 Japan; 6 Department of Oral Function & Anatomy, Okayama University Graduate School of Medicine, Dentistry and Pharmaceutical Sciences, 2-5-1 Shikata-cho, Kita-ku, Okayama, 700-0914 Japan

## Abstract

**Background:**

Recently, we reported that toll-like receptor (TLR)2 and TLR4 localized on the glomerular endothelium in the glomeruli of streptozotocin (STZ)-induced type 1 diabetic mice and high fat diet feed-induced type 2 diabetic mice, and that periodontal pathogen Porphyromonas gingivalis LPS (Pg-LPS) administration lowered the survival rate of diabetic mice. The present study aims to examine the effect of TLR4 blocking on the suppression of Pg-LPS-induced diabetic nephropathy.

**Methods:**

The survival rate and morphological/biochemical features for streptozotocin-induced diabetic mice with Pg-LPS and TLR4 blocker eritoran administration were investigated by reporter gene assay, urine and blood analysis, immunohistochemistry, and real time-PCR.

**Results and Conclusions:**

All of the diabetic mice administered Pg-LPS were euthanized within the survival period of almost all of the diabetic mice. The blood urea nitrogen and creatinine, expression of TLR2 and TGF-b, and type 1 collagen accumulation, in the diabetic mice increased significantly with the Pg-LPS administration. In spite of the limited TLR4 activation with Pg-LPS, the TLR4 blocker eritoran decreased blood urea nitrogen and creatinine, and raised the survival rate of the Pg-LPS-administered diabetic mice slightly. The high expression levels of TLR2, TGF-b, and type 1 collagen in Pg-LPS-administered diabetic mice decreased with eritoran. Nuclear STAT3 which enhances TLR2 expression was detected in the TLR2-expressing glomeruli of diabetic mice. The TLR2 and STAT3 gene expression increased by the Pg-LPS administration but decreased with eritoran. These may suggest that Pg-LPS-induced diabetic nephropathy is mainly dependent on TLR2 signaling on glomerular endothelial cells, and that TLR4 blocker eritoran may play a role to slow the progress of diabetic nephropathy.

## Background

Diabetes mellitus is a dysfunction of the glucose metabolism caused by an absence or insufficient production of insulin and gives rise to serious complications like diabetic nephropathy. Diabetic nephropathy is a serious complication characterized by renal failure with occlusion of the glomerular capillaries based on glomerulosclerosis: excess accumulation of extracellular matrix, glomerular basement membrane thickening, and increases in the mesangium composed of extracellular matrix proteins from mesangial cells. It has been thought that hydroxyl radicals and advanced glycation end products (AGE) like Nε-(carboxymethyl) lysine (CML), which are generated under a hyperglycemic environment, gives rise to oxidative stress and induces the production of various cytokines through the recognition of AGE [1–7]. However, there are individual differences in whether diabetic patients suffer from diabetic nephropathy and the factors that give rise to the differences are not well understood.

The renal metabolic recognition of AGE by the toll-like receptor (TLR) which is a sensor for pathogen-associated molecular patterns common to bacterial components has been suggested as one candidate for the occurrence of diabetic nephropathy because as at least TLR2 and TLR4 are clearly detected in blood when diabetic nephropathy is established, and the blood AGE elevates the TLR2 and TLR4 levels in monocytes and glomeruli in diabetic nephropathy [8–13]. It has been established that gram-positive bacterial components like lipoteichoic acid are recognized by TLR2 and that gram-negative bacterial components like lipopolysaccharide (LPS) are recognized by TLR4, and further that LPS from the periodontal pathogen *Porphyromonas gingivalis* binds both TLR2 and TLR4 [14–16]. The TLR ligand engagement activates the TLR regulatory gene MyD88 and NF-κB, and gives rise to the production of pro-inflammatory cytokines like tumor necrosis factor-α (TNF-α) and interleukin (IL)-6 which enhance tissue destruction. The TLR engagement also activates production of fibrogenic cytokines like the transforming growth factor-β (TGF-β) which allows mesangial cells around glomerular endothelial cells to produce extracellular matrix proteins playing a central role in tissue repair and glomerulosclerosis [17–21].

We recently reported that TLR2 and TLR4 localize on the glomerular endothelium in the glomeruli of STZ-induced type 1 diabetic mice and in high fat diet feed-induced type 2 diabetic mice [22, 23]. The detected levels of renal TLR2 and TLR4 mRNA were higher in diabetic mice than in non-diabetic mice. In that study of the effects of the periodontal pathogen *P. gingivalis* lipopolysaccharide (LPS) which is a ligand of TLR2 and TLR4 in diabetic nephropathy, all diabetic mice subjected to repeated LPS administrations were euthanized within the survival period of all of the diabetic mice not administered LPS and of the survival period of all of the non-diabetic LPS-administered mice. The LPS administration promoted the production of urinary protein, the accumulation of type 1 collagen in the glomeruli, and increases in the IL-6, TNF-α, and TGF-β in glomeruli of the renal cortex of the diabetic mice. Components from gastrointestinal bacteria are metabolized in the liver while microorganisms from the oral cavity and pharynx directly enter the kidney through the systemic circulation. Severe periodontitis commonly gives rise to bacteremia. Blood TLR ligands from periodontal pathogens like *P. gingivalis* may accumulate in the glomeluri and induce chronic renal inflammation, tissue repair, and glomerulosclerosis [23–29]. In streptozotocin induced-type 1 diabetic mice (STZ mice), TLR4 promotes renal injury and interstitial fibrosis while all of these are suppressed in TLR4 KO-STZ mice. The TLR blockages may prevent the progress of diabetic nephropathy since albuminuria and glomerular hypertrophy in the TLR4 KO-STZ mice is less pronounced than in the wild type-STZ mice [30–35].

*Porphyromonas gingivalis* is an important microorganism in causing periodontitis [36]. *Porphyromonas gingivalis* produces very large amounts of LPS in the outer membrane which leads to periodontal tissue destruction and tooth loss. The biological potential of *P. gingivalis* LPS is lower than the LPS from *Escherichia coli* but *P. gingivalis* LPS which has entered the systemic circulation induces several immunological events.

The present study aims to examine the effect of TLR4 blocking to the progress of diabetic nephropathy promoted by *P. gingivalis* LPS.

## Methods

This reports an animal study performed to achieve the project goals of determining the survival rate of a streptozotocin (STZ)-induced diabetic mouse with periodontal pathogen *P. gingivalis* (Pg)-LPS and TLR4 blocker eritoran (Eisai Inc., Andover, MA, USA) administration. Further, a morphological investigation of details of the promotion of diabetic nephropathy arising with the Pg-LPS administration. The studies here used control and experimental groups with nine mice in each group (2/cage). The manuscript was prepared following the ARRIVE guidelines.

### Animals

The experimental protocol for the animal use was reviewed and approved by the Animal Experiment Committee of Fukuoka Dental College in accordance with the principles of the Helsinki Declaration. Breeding and experiments were performed in a room with a 100% controlled atmosphere which had passed an examination for bacteria and is located in the Fukuoka Dental College Animal Center. Mice grew normally and lived healthily under conventional atmosphere conditions with normal feeding in cages and rooms with temperature (22 °C) and humidity (55%) completely controlled. The mice were housed with an inverse 12 h day-night cycle with lights on from 7:00 p.m.

Humane endpoints were used in the experiments as a rapid and accurate method for assessing the health status of the mice, that is, mice with loss of the ability to ambulate (inability to access food or water) were euthanized by induction anesthesia (1 l/min of 2% isoflurane mixed with 30% oxygen and 70% nitrous oxide with an anaesthetic apparatus) followed by cervical dislocation and intraperitoneal injections with sodium pentobarbital (10 ml/kg, Nembutal, Abbott Laboratories, North Chicago, IL) or 3.5% chloral hydrate (10 ml/kg, trichloroacetaldehyde monohydrate, Kanto Chemical, Tokyo, Japan) in the saline.

The streptozotocin (STZ)-injected closed line ICR mice (Japan Clea Inc., Osaka, Japan) were used as the diabetic model [25]. Six-week-old male ICR mice were given a single intraperitoneal injection of STZ (200 mg/kg body weight) (Sigma, St. Louis) in a 0.05 M citric acid buffer at pH 4.5 (20 mg/ml). The non-diabetic controls were ICR mice intraperitoneally administered with 0.05 M citric acid buffer only. The injection was conducted under inhalation anesthesia with 2% isoflurane. Carprofen (7.5 mg/kg; Wako Pure Chemical Industries, Ltd., Osaka, Japan) was used as pre-injection for preemptive analgesia and also in post-injections every 12–24 h. The animal condition was checked every 8 h for the first 2 days after the injection, and every 12 h thereafter. The blood glucose was checked once a week after the injection by measurements of the blood glucose using a Glutest Sensor (Sanwa Kagaku Kenkyusyo CO., LTD., Nagoya, Japan). One week after the STZ injection diabetes was confirmed and ICR mice with blood glucose above 600 mg/dl for 4 months were designated as STZ-induced type 1 diabetic mice. All mice were euthanized by induction anesthesia with 2% isoflurane followed by cervical dislocation at the end of the designated period of the experiments, and the mouse tissue was collected.

### *Porphyromonas gingivalis* lipopolysaccharide (Pg-LPS)

The Pg-LPS as purchased from Wako (Tokyo, Japan) was used in the in vivo experiments. Other Pg-LPS were tested for a comparative examination of the Wako Pg-LPS activity to TLR2 and TLR4: LPS obtained from *P. gingivalis* was cultured with hemin at high and low concentrations (Darveau laboratory) because of the requirement of hemin as an iron source for the growth of *P. gingivalis*, Pg-LPS purchased from Invivogen (San Diego, California, USA), and LPS consisting of tetra-acylated lipid A structures with low induction of the host response (Pg-LPS1435, Darveau laboratory). The *E. coli* LPS (*E. coli* LPS 0111:B4, TLR4 agonist, Invivogen) and synthetic lipoprotein tripalmitoyl-*S*-glyceryl-cysteine (Pam3CSK4; TLR1/2 agonist, Invivogen) were used as controls. The Pam3CSK4 was used after an overnight incubation at 37 °C with lipase from a *Burkholderia* sp. (Sigma-Aldrich, St. Louis, MO) to exclude lipid.

### Reporter gene assay for TLR activation

Experiments were carried out five times as previously reported [37]. Here, HEK293 cells were cotransfected with plasmids encoding NF-κB-dependent firefly luciferase reporter, β-actin promoter-dependent *Renilla* luciferase reporter, and human TLR2/TLR1 (cotransfected with plasmid encoding human mCD14) or human TLR4 (cotransfected with plasmid encoding human MD-2). Luciferase activity was assayed using a dual luciferase assay reporter system (Promega, Madison, WI) on the HEK293 cells cultured with LPS. The NF-κB activity was measured as the ratio of NF-κB-dependent firefly luciferase activity to β-actin promoter-dependent Renilla luciferase activity, which served as an internal standard.

### *Porphyromonas gingivalis* lipopolysaccharide (Pg-LPS) administration to diabetic mice

To investigate the survival rate of the diabetic mice with Pg-LPS, a 100-µl volume (100 µg, 1 mg/ml) of Pg-LPS (3 mg/kg, LD50 = 30 mg/kg body weight, Wako) was administered repeatedly under the buccal mucosa once a week to the STZ-induced diabetic mice which had shown blood glucose levels above 600 mg/dl for 4 months [26, 27]. To investigate the effect of the TLR4 blocker eritoran (83142-1:2013-0162:eritoran/E5564, Eisai Inc. USA) on the survival rate of the diabetic mice administered Pg-LPS, a 100-µl volume (100 µg, 1 mg/ml) of eritoran (LPS-dependent blood vessel activation inhibitory concentration in the mouse aorta organ culture, Eisai) was subcutaneously injected into the back skin of mice three times (0, 24, and 48 h after the Pg-LPS administration) a week. The injection was conducted under inhalation anesthesia with 2% isoflurane. Carprofen (7.5 mg/kg; Wako) was used as pre-injection for preemptive analgesia and post-injection every 12–24 h. The animal condition was checked every 8 h for the first 2 days after the injection, and every 12 h thereafter. The urine of the ICR mice, the ICR mice with repeated LPS administrations, the STZ-induced diabetic mice, and the STZ-induced type 1 diabetic mice with repeated LPS administrations, was analyzed for sugar, protein, and bleeding by urine reagent strips (Uriace, Terumo Corporation, Tokyo, Japan) once a week from the 1st LPS administration and at the humane endpoints as described above (27th week). The blood of the experimental mice was collected from the tail vein and analyzed for blood urea nitrogen (BUN) and creatinine (CRE) by Kyudo Co., LTD (Tosu, Japan) once a week from the 1st LPS administration and at the humane endpoints described above. All mice were euthanized by induction anesthesia with 2% Isoflurane followed by cervical dislocation at the end of the designated period of the experiments, and the mouse tissue was collected.

### Immunohistochemistry

Frozen 10 μm mouse kidney tissue sections cut in a cryostat were placed on slide glass. The sections were fixed in 100% methanol for 5 min at −20 °C, treated with 0.1% goat serum for 30 min at 20 °C, and then treated for 8 h at 4 °C with PBS containing 0.1% goat serum and the following primary antibodies (1 μg/ml): hamster monoclonal anti-mouse podoplanin (AngioBio Co., Del Mar, CA) as a podocyte marker, rat monoclonal anti-mouse TLR2 (R&D Systems Inc., Minneapolis, MN), rabbit polyclonal anti-mouse TGF-β (Abcam plc., Cambridge, UK), rabbit polyclonal anti-type 1 collagen (Abcam), and rabbit polyclonal anti-mouse STAT3 (Abcam). After the treatment with primary antibodies the sections were washed three times in PBS for 10 min and immunostained for 0.5 h at 20 °C with 0.1 μg/ml of second antibodies: Alexa Fluor (AF) 488 or 568-conjugated goat anti-hamster, goat anti-rabbit, or goat anti-rat IgGs (Probes Invitrogen Com., Eugene, OR). The immunostained sections were mounted in 50% polyvinylpyrrolidone solution and examined by fluorescence microscopy (BZ-8100, Keyence Corp., Osaka, Japan) or confocal laser-scanning microscopy (LSM710, Carl Zeiss, Jena, Germany) with an 63× oil Plan Apochromatic objective lens (numerical aperture 1.3×).

### Measurements of the immunostained areas of tissue sections

Areas immunostained by anti-podoplanin, anti-TLR2, anti-TGF-β, and anti-type 1 collagen were measured around different glomeruli (10/section) in laser-scanned microscopic images at 819× magnification by ImageJ (National Institutes of Health, Bethesda, MD). The relative expressed amounts of anti-TLR2, anti-TGF-β, and anti-type 1 collagen were expressed by the means of the ratio: area of anti-TLR2, anti-TGF-β, or anti-type 1 collagen in a glomerulus/area of a glomerulus within podoplanin-positive podocytes.

### Reverse transcription (RT)-PCR

Renal cortex tissue was peeled away from the kidney within a 5 mm^2^ by an 18-gauge needle under a stereoscopic microscope. The total RNA extraction from the tissue was performed with a QIAshredder column and an RNeasy kit (Qiagen, Inc., Tokyo, Japan). Contaminating genomic DNA was removed using DNAfree (Ambion, Huntingdon, UK), and the RT was performed on 30 ng of total RNA, followed by 30 cycles of PCR for amplification using the Ex Taq hot start version (Takara Bio Inc., Otsu, Japan) with 50 pM of primer sets for mouse β-actin, TLR2, and STAT3 mRNAs (Table 1), where the specificities had been confirmed by the manufacturer (Sigma-Genosys Ltd., Cambridge, UK). The RT-PCR products were separated on 2% agarose gel (NuSieve; FMC, Rockland, ME, USA) and visualized by Syber Green (Takara). The correct size of the amplified PCR products was confirmed by gel electrophoresis and amplification of accurate targets was confirmed by sequence analysis.

**Table 1:** Sequence of primers

Protein	bp	Upper (5′–3′)	Lower (5′–3′)
β-Actin	411	GTTCTACAAATGTGGCTGAGGA	ATTGGTCTCAAGTCAGTGTACAG
TLR2	210	TGTTTCTGAGTGTAGGGGCTTCA	ACATGACAGAGACTCCTGAGCAG
STAT3	224	AACATTCTGGGCACGAACACAA	AAGGAGTGGGTCTCTAGGTCAATC

### Statistics

All experiments were repeated five times, and data are expressed as the mean + SD. The statistical significance of differences (p < 0.05) was determined by the two-tailed unpaired Student’s t test and one-way ANOVA with STATVIEW 4.51 software (Abacus concepts, Calabasas, CA, USA). Mean values were calculated with standard deviation (STDEV) unless stated otherwise.

## Results

### Activation of TLR2 and TLR4 by Pg-LPS

**Fig. 1:**
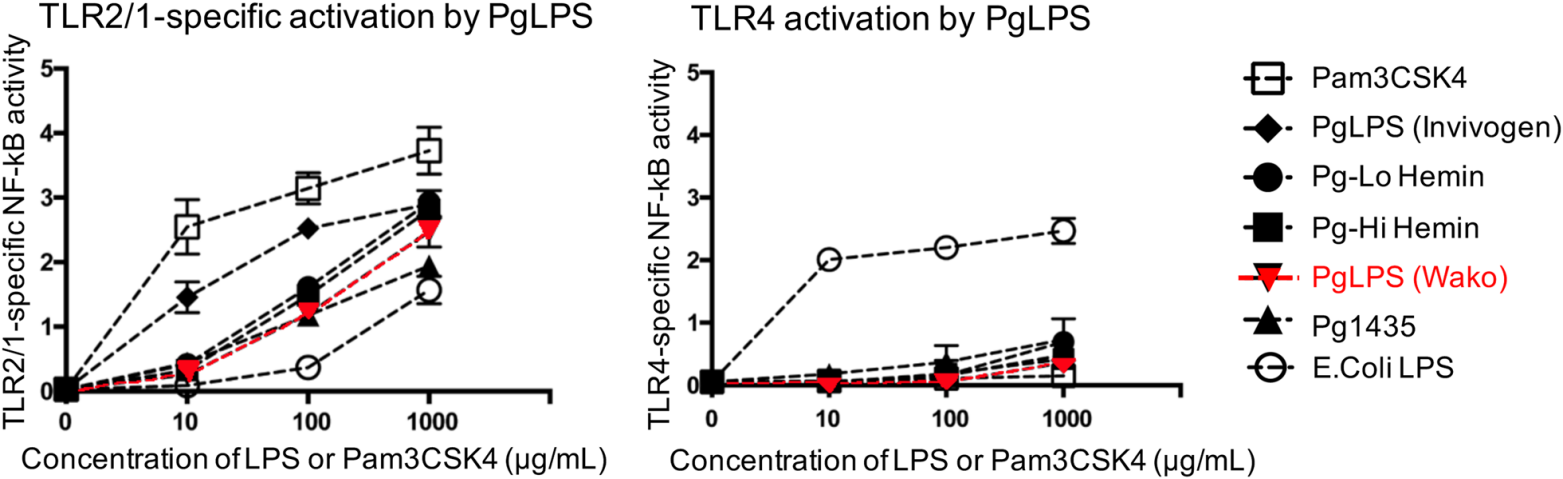
TLR activation by *P. gingivalis* lipopolysaccharide (Pg)-LPS. The NF-κB activity on reporter and TLR-cotransfected HEK293 cells cultured with LPS was measured as the ratio of NF-κB-dependent firefly luciferase activity to β-actin promoter-dependent Renilla luciferase activity. For the TLR2/1-specific NF-κB activation (left panel), the synthetic tripalmitoyl-*S*-glyceryl-cysteine (Pam3CSK4; TLR1/2 agonist, InvivoGen) showed the highest activity among all the chemicals tested. The Pg-LPS from Invivogen, Pg-LPS from Wako, and LPS obtained from *P. gingivalis* cultured with hemin at high and low concentrations (Pg-Lo Hemin and Pg-Hi Hemin) were lower and of similar intensity. The *E. coli* LPS (*E. coli* LPS) and LPS consisting of tetra-acylated lipid A structures (Pg1435) showed the lowest activity among all the chemicals tested for the host response via TLR2/1. For the TLR4-specific NF-κB activation (right panel), *E. coli* LPS (*E. coli* LPS) showed the highest activity among all the chemicals tested. The Pg-LPS from Invivogen, Pg-LPS from Wako, LPS obtained from *P. gingivalis* cultured with hemin at high and low concentrations (Pg-Lo Hemin and Pg-Hi Hemin), and LPS consisting of tetra-acylated lipid A structures (Pg1435) form an extremely low activity group, and the synthetic tripalmitoyl-*S*-glyceryl-cysteine (Pam3CSK4) also showed little activity. Experiments were repeated five times and statistically analyzed. There were significantly differences between Pam3CSK4 and others in TLR2/1 activation, and between *E. coli* LPS and others in TLR4 activation by ANOVA

For the TLR2 activation (Fig. 1), the synthetic TLR1/2 agonist Pam3CSK4 showed the highest activity among all the chemicals tested. The Pg-LPS from Invivogen, Pg-LPS from Wako, and Pg-LPS from *P. gingivalis* cultured with hemin, formed a group with activities higher than *E. coli* LPS and Pg-LPS1435 with tetra-acylated lipid A structures (Fig. 1). For the TLR4 activation, *E. coli* LPS showed the highest activity in all the chemicals tested. The Pg-LPS from Invivogen, the Pg-LPS from Wako, the Pg-LPS from *P. gingivalis* cultured with hemin, and the LPS with tetra-acylated lipid A structures formed an extremely low activity group, and the Pam3CSK4 also showed little activity.

### Influence of Pg-LPS on the STZ-induced type 1 diabetic mice

**Fig. 2:**
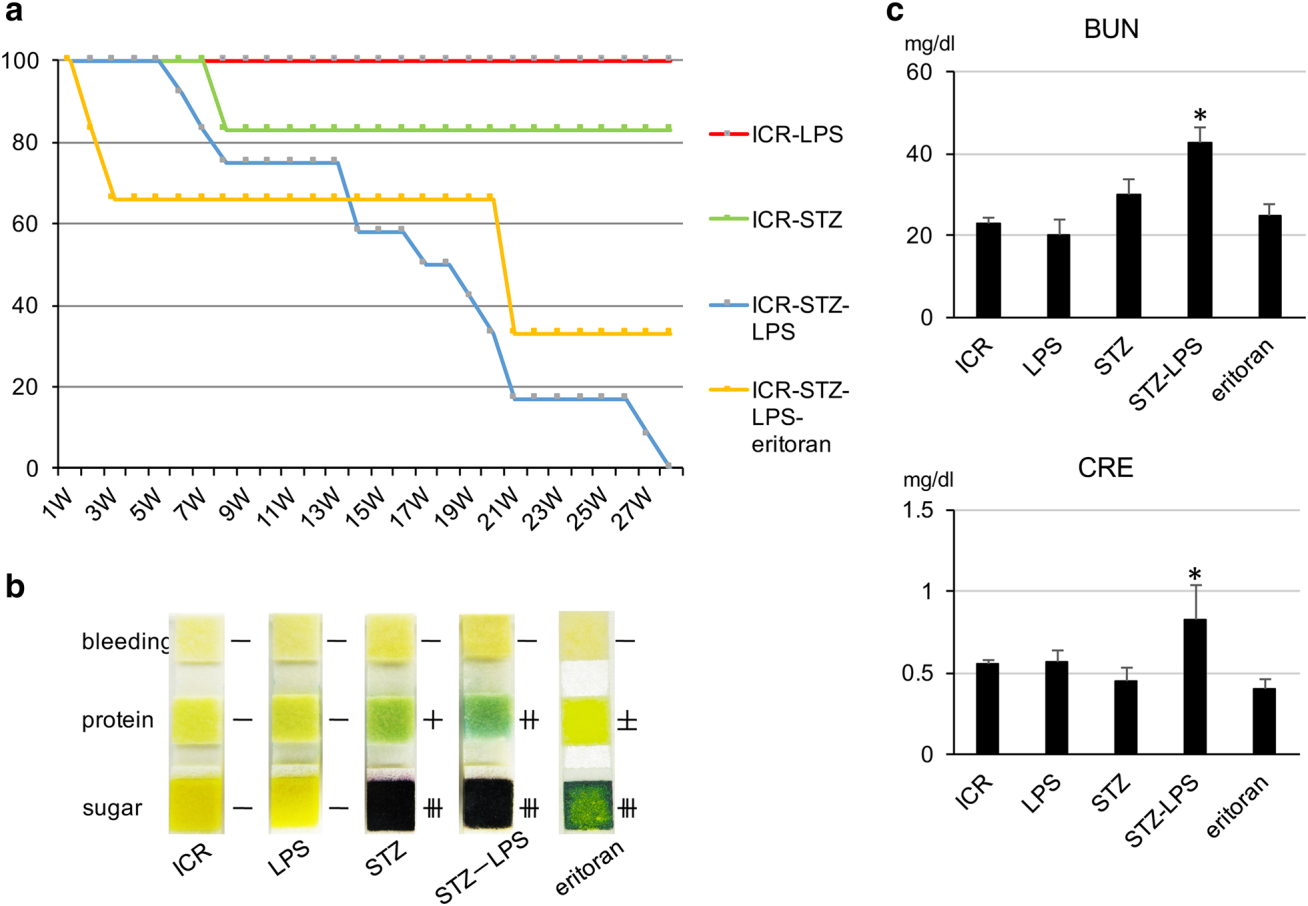
Influence of Pg-LPS on the STZ-induced diabetic mice. The survival rate of the STZ-induced diabetic mice with Pg-LPS and the effect of the TLR4 blocker eritoran on the survival rate of the diabetic mice administered Pg-LPS were investigated. **a** Survival curve of the STZ-induced type 1 diabetic ICR mice with repeated administrations of Pg-LPS under the buccal mucosa. All of the nine diabetic mice administered Pg-LPS (blue curve, ICR-STZ-LPS) were euthanized within the survival period of all of the non-diabetic mice administered Pg-LPS (red curve, ICR-LPS) as did almost all of the diabetic mice not administered Pg-LPS (green curve, ICR-STZ). Diabetic mice administered both Pg-LPS and eritoran showed a slightly higher survival rate than those without eritoran administration (orange curve, ICR-STZ-LPS-eritoran). **b** Urinalysis of the STZ-induced type 1 diabetic ICR mice administered Pg-LPS, at the 27th week of survival. Urine reagent strips show sugar, protein and bleeding in the urine of non-diabetic ICR mice (ICR), non-diabetic ICR mice administered Pg-LPS (LPS), STZ-induced type 1 diabetic ICR mice not administered Pg-LPS (STZ), diabetic mice administered Pg-LPS (STZ-LPS), and diabetic mice administered both Pg-LPS and eritoran (eritoran). Extensive amounts of urinary sugar were observed in the diabetic mice (STZ) and the diabetic mice administered Pg-LPS (STZ-LPS), and the urinary protein content was higher in the diabetic mice administered Pg-LPS (STZ-LPS) than in the diabetic mice not administered LPS (STZ). Eritoran administration resulted in a lower urinary protein content in the surviving diabetic mice administered Pg-LPS. **c** Blood analysis for the STZ-induced type 1 diabetic ICR mice administered Pg-LPS. Blood from the tail vein was analyzed for blood urea nitrogen (BUN) and creatinine (CRE) at the 27th week of survival. Both BUN and CRE were significantly higher in the diabetic mice administered Pg-LPS (STZ-LPS) than in the diabetic mice not administered Pg-LPS (STZ) and other mice. There appears to be decreases in BUN and CRE in the surviving diabetic mice administered Pg-LPS and eritoran. Data are expressed as means + SD. *Significantly different in one-way ANOVA (p < 0.05)

All of the STZ-induced type 1 diabetic mice administered Pg-LPS were euthanized within the survival period of all of the non-diabetic mice administered Pg-LPS and of almost all of the diabetic mice not administered Pg-LPS (Fig. 2). Eritoran induced a slight degree of remission in the diabetic mice administered Pg-LPS. In the urinalysis, extensive amounts of urinary sugar were observed in the diabetic mice and the diabetic mice administered Pg-LPS, and the urinary protein level was higher in the diabetic mice administered Pg-LPS than in the diabetic mice not administered LPS, at the humane endpoints of survival (27th week) (Fig. 2). There appears to be a lower urinary protein level in the surviving diabetic mice administered Pg-LPS and eritoran. In the blood analysis, both urea nitrogen and creatinine amounts were significantly higher in the diabetic mice administered Pg-LPS than in the diabetic mice not administered Pg-LPS, also at the humane endpoints of survival (27th week) (Fig. 2). There was a slight recovery with eritoran in both the urea nitrogen and creatinine levels of the surviving diabetic mice administered Pg-LPS.

### Expression of TLR2, TGF**-**β, and type 1 collagen in the glomeruli of STZ-induced type 1 diabetic mice administered Pg-LPS

**Fig. 3:**
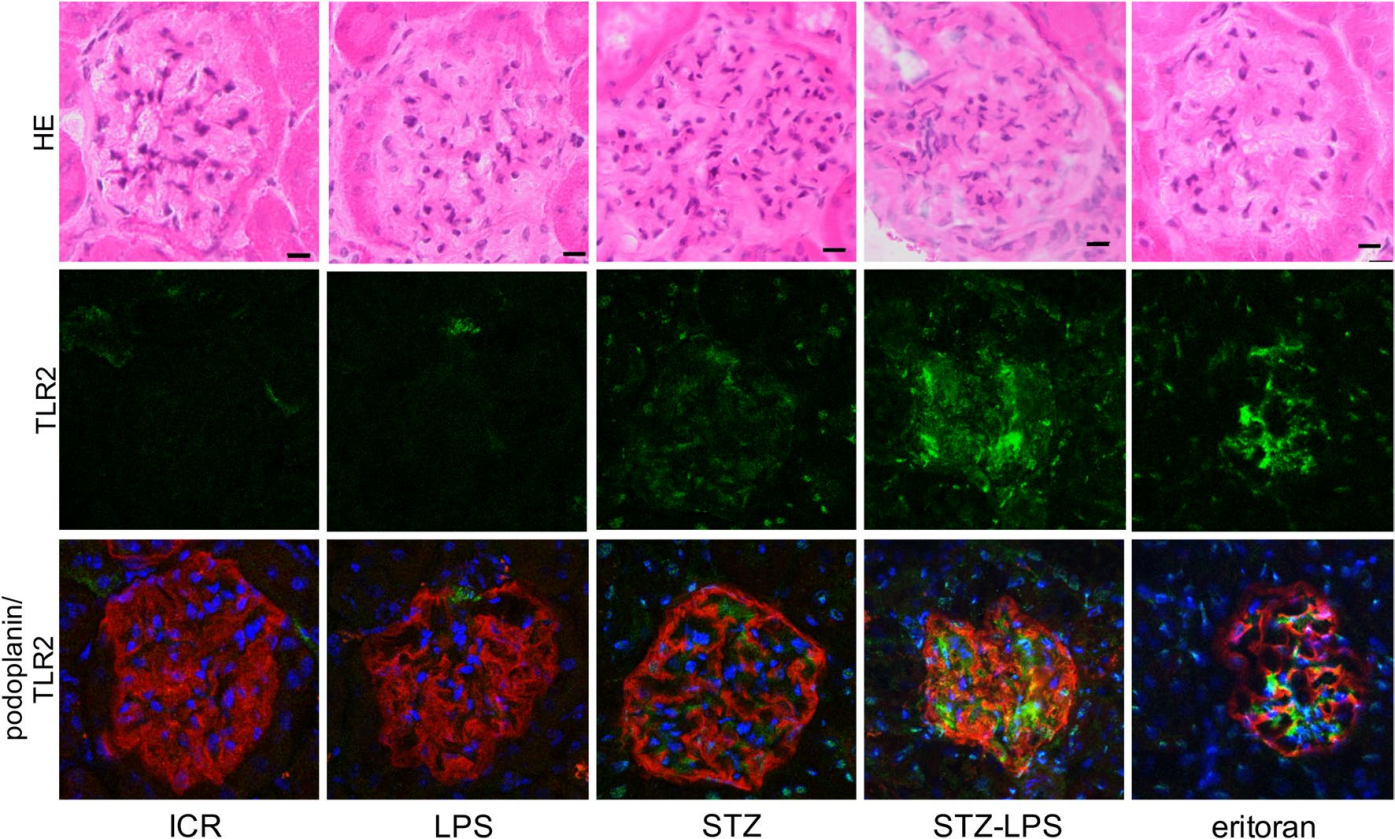
Expression of TLR2 in the STZ-induced type 1 diabetic mouse glomeruli. HE staining (upper lane), immunostaining for TLR2 (green, middle lane), and merged (lower lane) immunostaining for TLR2 (green) and podoplanin (red), with DAPI staining of nuclei (blue). The TLR2 expression locates between podocytes immunostained by anti-podoplanin. Little expression of TLR2 is observed in the non-diabetic ICR mice (ICR) and non-diabetic ICR mice administered Pg-LPS (LPS), but TLR2 is observed in the STZ-induced type 1 diabetic mouse glomeruli. The expression level of TLR2 was higher in the diabetic mice administered Pg-LPS (STZ-LPS) and the level slightly lower in the diabetic mice administered both Pg-LPS and eritoran (eritoran). Bars: 20 μm

**Fig. 4:**
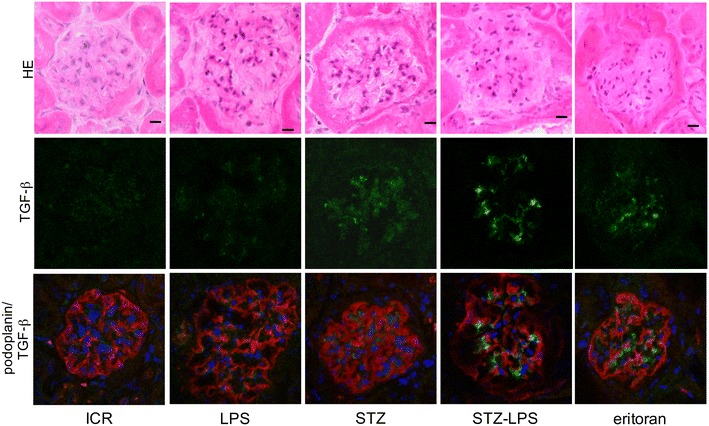
Expression of TGF-β in the STZ-induced type 1 diabetic mouse glomeruli. HE staining (upper lane), immunostaining for TGF-β (green, middle lane), and merged (lower lane) immunostaining for TGF-β (green) and podoplanin (red), with DAPI staining of nuclei (blue). The TGF-β expression locates between podocytes immunostained by anti-podoplanin. Expression of TGF-β is little observed in the non-diabetic ICR mice (ICR) and non-diabetic ICR mice administered Pg-LPS (LPS), but TGF-β is observed in the STZ-induced type 1 diabetic mouse glomeruli. The expression level of TGF-β was higher in the diabetic mice administered Pg-LPS (STZ-LPS) and the level was slightly lower in the diabetic mice administered both Pg-LPS and eritoran (eritoran). Bars: 20 μm

**Fig. 5:**
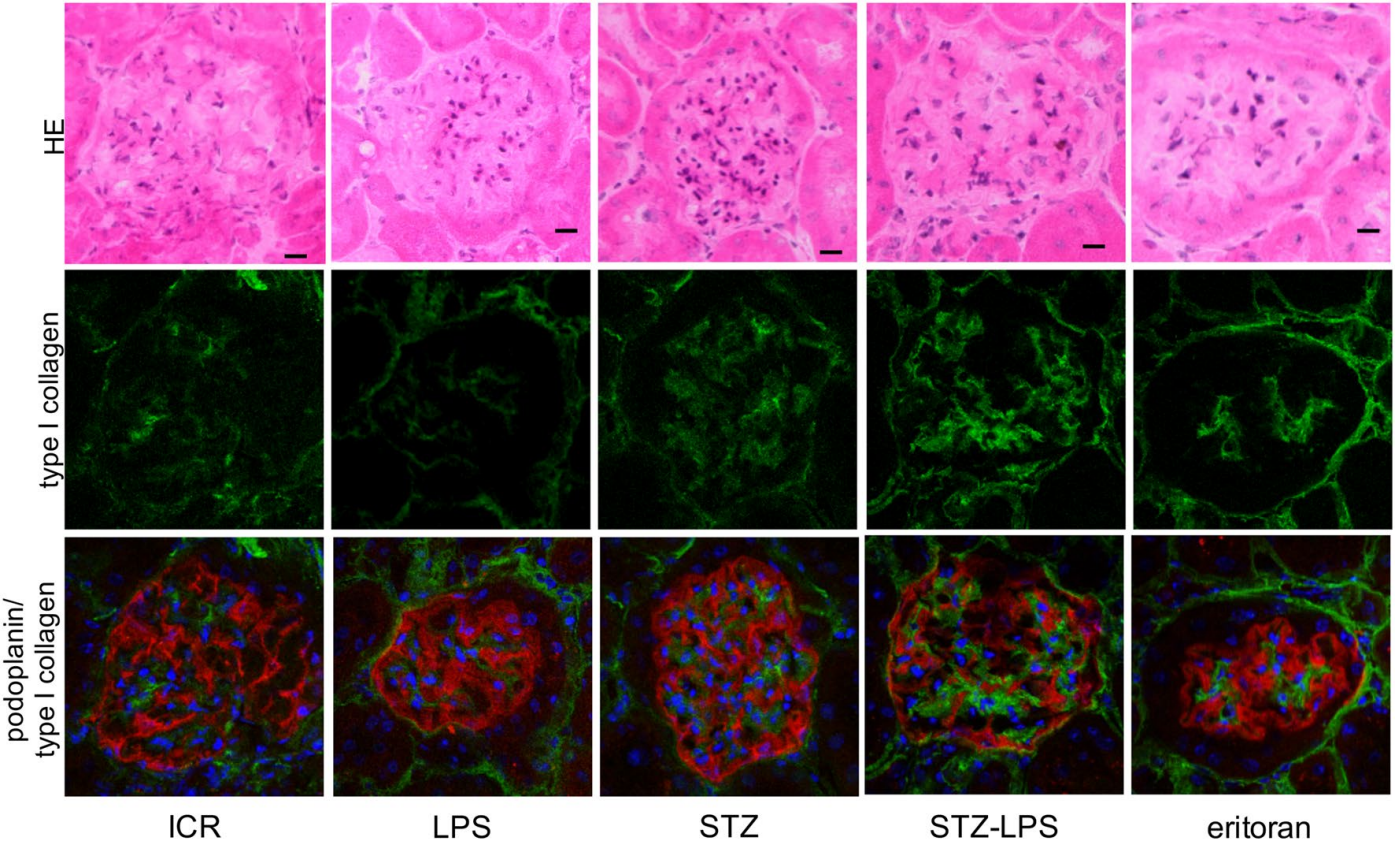
Expression of type 1 collagen in the STZ-induced type 1 diabetic mouse glomeruli. HE staining (upper lane), immunostaining for type 1 collagen (green, middle lane), and merged (lower lane) immunostaining for type 1 collagen (green) and podoplanin (red), and DAPI staining of nuclei (blue). The type 1 collagen expression locates between podocytes immunostained by anti-podoplanin. Expression of type 1 collagen is observed in the non-diabetic ICR mice (ICR) and non-diabetic ICR mice administered Pg-LPS (LPS), and is stronger in the STZ-induced type 1 diabetic mouse glomeruli. The expression of type 1 collagen is stronger in the diabetic mice with Pg-LPS (STZ-LPS) than in the diabetic mouse not administered Pg-LPS, and the level is slightly lower in the diabetic mice administered with Pg-LPS and eritoran (eritoran). Bars: 20 μm

**Fig. 6:**
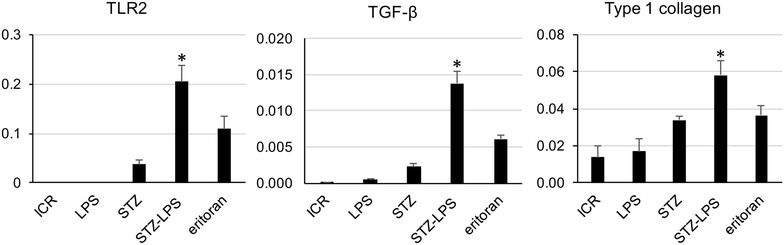
Expressied amounts of TLR2, TGF-β, and type 1 collagen in the STZ-induced type 1 diabetic mouse glomeruli. Areas immunostained by anti-podoplanin, anti-TLR2, anti-TGF-β, and anti-type 1 collagen were measured around different glomeruli (10/section) in laser-scanned microscopic images at 819x magnification by ImageJ. The relative expressed amounts of anti-TLR2, anti-TGF-β, and anti-type 1 collagen were expressed by the mean of the ratio: the area of anti-TLR2, anti-TGF-β, and anti-type 1 collagen in a glomerulus/area of a glomerulus within the podoplanin-positive podocytes. Expression of TLR2 and TGF-β in glomeruli was almost fully absent in the non-diabetic ICR mice (ICR) and in the non-diabetic ICR mice administered Pg-LPS (LPS), but TLR2 and TGF-β was detected in the STZ-induced type 1 diabetic mice (STZ). Expressed amounts of TLR2 and TGF-β in glomeruli were much higher in the diabetic mice administered Pg-LPS (STZ-LPS) and the level was lower in the diabetic mice administered both Pg-LPS and eritoran (eritoran). Type 1 collagen was detected in the non-diabetic ICR mice (ICR) and non-diabetic ICR mice administered Pg-LPS (LPS), and the expressed amount was lower in the STZ-induced type 1 diabetic mice (STZ). The type 1 collagen accumulation in the glomeruli was much higher in the diabetic mice administered Pg-LPS (STZ-LPS) and the level was lower in the eritoran-administered diabetic mice with Pg-LPS (eritoran). Data are expressed as means + SD. *Significantly different in the one-way ANOVA (*p* < 0.01)

Expression of TLR2 was very limited in the non-diabetic mice and the non-diabetic mice administered Pg-LPS, but was present in STZ-induced type 1 diabetic mouse glomeruli (Fig. 3). The expression level of TLR2 was stronger in the diabetic mice administered Pg-LPS and slightly lower in the Pg-LPS-administered diabetic mice also administered eritoran. Expression of TGF-β was very limited in the non-diabetic mice and the non-diabetic mice administered Pg-LPS, but it was present in the diabetic mouse glomeruli (Fig. 4). The expression of TGF-β was stronger in the diabetic mice administered Pg-LPS and slightly weaker in the diabetic mice administered both Pg-LPS and eritoran. Accumulation of type 1 collagen was observed in the non-diabetic mice and in the non-diabetic mice administered Pg-LPS, and was higher in the diabetic mouse glomeruli (Fig. 5). Accumulation levels of type 1 collagen were stronger in the diabetic mice administered Pg-LPS than in the diabetic mice not administered Pg-LPS, and the expression was slightly weaker in diabetic mice administered both the eritoran and Pg-LPS. In the quantitative analysis of the immunostaining by ImageJ, the expression of TLR2 and TGF-β in the glomeruli was close to nil in the non-diabetic mice and in the non-diabetic mice administered Pg-LPS, but TLR2 and TGF-β were detected in the diabetic mice (Fig. 6). Expressed amounts of TLR2 and TGF-β in glomeruli increased very much in the diabetic mice administered Pg-LPS and the level decreased in the diabetic mice administered both Pg-LPS and eritoran. Type 1 collagen was observed in the non-diabetic ICR mice and non-diabetic ICR mice administered Pg-LPS, and the expression was stronger in the diabetic mice. The type 1 collagen accumulation in the glomeruli was very high in the diabetic mice administered Pg-LPS and lower in the diabetic mice administered both Pg-LPS and eritoran.

### Expression of STAT3 on the TLR2-positive glomeruli in the STZ-induced type 1 diabetic mice

**Fig. 7:**
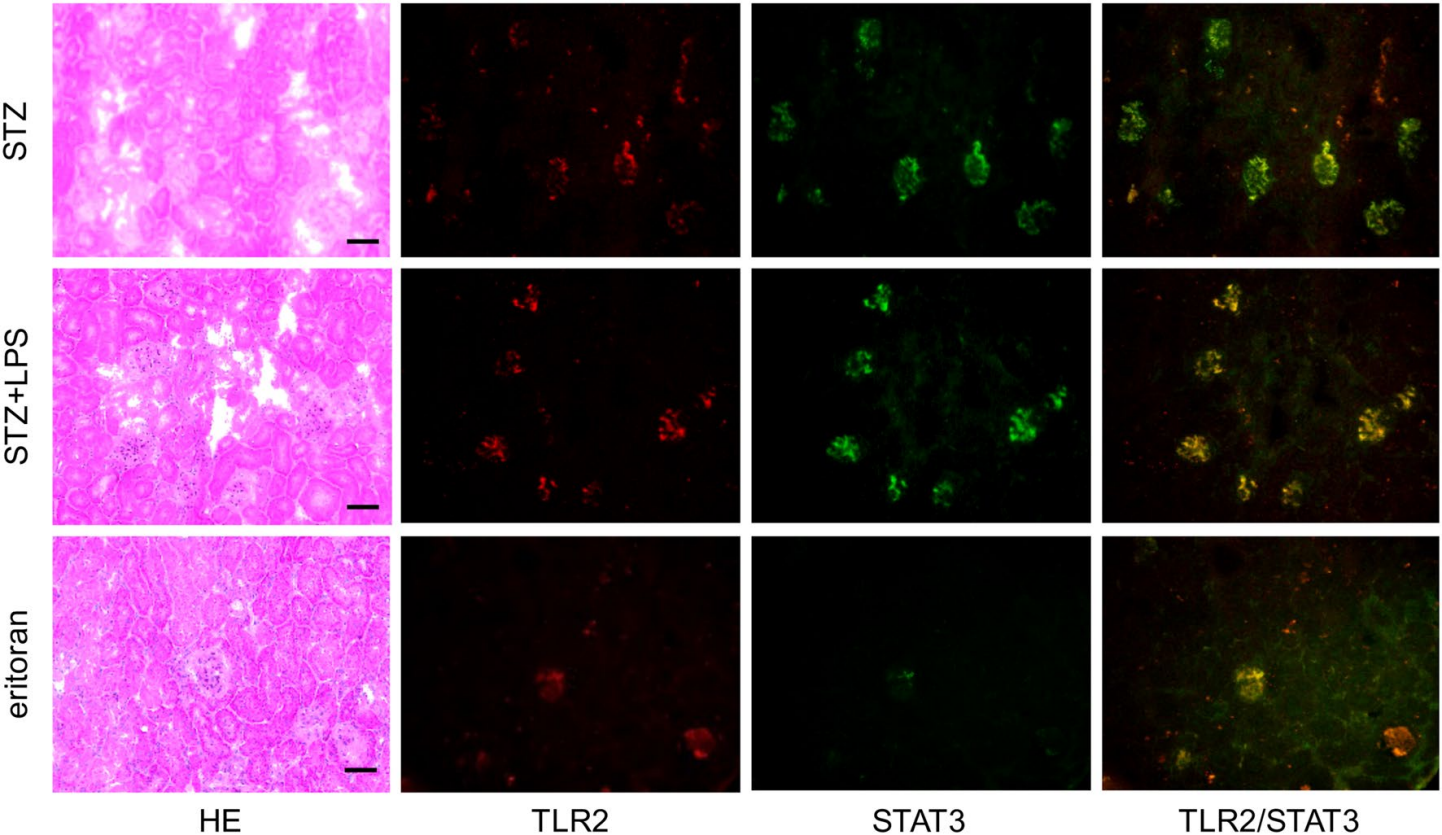
Distribution of STAT3-positive cells in the diabetic mouse kidneys. The STAT3 was detected in the TLR2-positive glomeruli of the STZ-induced type 1 diabetic mice (STZ) and in the diabetic mice administered Pg-LPS (STZ-LPS). The number of glomeruli with both TLR2 and STAT3 expression is larger in the diabetic mice administered Pg-LPS than in the diabetic mice not administered Pg-LPS. There are also some glomeruli with TLR2 and STAT3 expression in the eritoran-administered diabetic mice administered Pg-LPS (eritoran). Bars: 100 μm

**Fig. 8:**
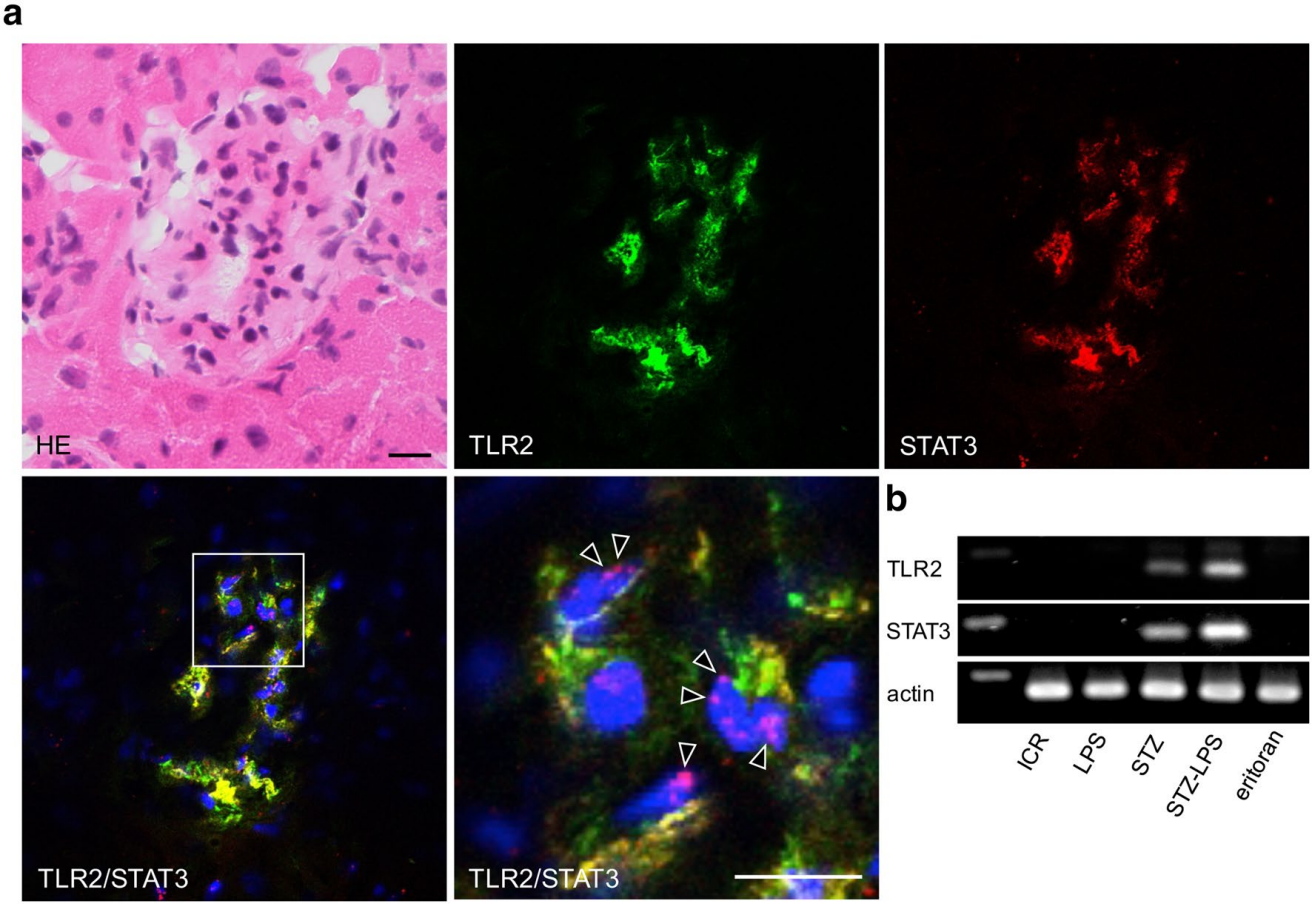
Expression of STAT3 in diabetic mouse glomeruli. **a** Localization of TLR2 and STAT3 in the diabetic mouse glomeruli. In the laser-scanning confocal microscopy, the regions expressing TLR2 coincided with the regions expressing STAT3, and nuclear localization of STAT3 is observed in the nuclei of both TLR2 and STAT3-expressed cells (magnified area of the merged image, arrowheads). Bars: 20 μm. **b** Tissue PCR for TLR2 and STAT3 mRNA of the diabetic mouse renal cortex with Pg-LPS and eritoran administration. The mRNAs of TLR2 and STAT3 were detected in the STZ-induced type 1 diabetic ICR mice (STZ), and the expressed levels were higher in the diabetic mice with Pg-LPS (STZ-LPS). The mRNAs of STAT3 and TLR2 were not detectable in the non-diabetic mice (ICR), non-diabetic mice administered Pg-LPS (LPS), or diabetic mice administered both Pg-LPS and eritoran (eritoran)

The STAT3 was detected in the TLR2-positive glomeruli in the STZ-induced type 1 diabetic mice and in the diabetic mice administered Pg-LPS (Fig. 7). The number of glomeruli with both TLR2 and STAT3 expression is stronger in the diabetic mice administered Pg-LPS than in the diabetic mice not administered Pg-LPS. There were also some glomeruli with TLR2 and STAT3 expression in the diabetic mice administered with Pg-LPS and eritoran. However, STAT3 was not detected in the TLR2-negative glomeruli of the non-diabetic mice and non-diabetic mice administered Pg-LPS (not shown). In the laser-scanning confocal microscopic images (Fig. 8), the regions expressing TLR2 coincided with the regions expressing STAT3, and localization of STAT3 to the nuclei was observed in the nuclei of both STAT3 and TLR2-expressing cells. In the tissue PCR analysis of the diabetic mouse renal cortex (Fig. 8), the mRNAs of TLR2 and STAT3 were detected in the diabetic mice, and the expression levels increased in the diabetic mice administered Pg-LPS. The mRNAs of STAT3 and TLR2 were not detected in the non-diabetic mice, non-diabetic mice administered Pg-LPS, or in the diabetic mice administered Pg-LPS and eritoran.

## Discussion

### Dual features of Pg-LPS in TLR activation

The periodontal pathogen *P. gingivalis* is a bacterium which produces compounds including LPS, which is a cause of periodontitis and other diseases. It has been reported that the risk of heart attack and cerebrovascular diseases are significantly larger in groups with severe periodontal infections than in groups with mild periodontal disease, and that *P. gingivalis* induces platelet aggregation and promotes atherosclerosis [38–41]. The periodontal pathogen, *P. gingivalis*, is unique in that it produces LPS that is capable of engaging both TLR2 and TLR4 [36]. The Pg-LPS with lipid A structural variants, designated Pg-LPS1435/1449, show activity as TLR4 antagonists while what is designated Pg-LPS1690 and wild-type *E. coli* LPS are TLR4 agonists [42]. The lipid A phosphate determines lipopolysaccharides (LPS) as TLR4 agonists or antagonists. The phosphate position of lipid A is a key feature for determining the strikingly distinct TLR4 responses, inducing both health or disease in different situations. *Bacteroides thetaiotaomicron* which promotes intestinal health produces TLR4-stimulatory lipid A which has a 1-phosphate while *P. gingivalis* which is associated with the chronic oral inflammatory disease periodontitis produces TLR4-evasive lipid A containing a 4′-phosphate component [43]. The intrinsically weak TLR4 activity observed for Pg-LPS is due to the modification of the LPS lipid A structure by lipid A phosphatases resulting in functionally inert or antagonist structures [44]. *Porphyromonas gingivalis* with LPS consists of non-phosphorylated lipid A able to evade host defense systems via TLR4 sensing by endogenous phosphatase. Lipid A 1-phosphatase is suppressed by haemin which is an important nutrient for *P. gingivalis* in the gingival sulcus. Under conditions with high concentrations of haemin, *P. gingivalis* produces LPS with low lipid A 1-dephosphorylation which acts as a TLR4 antagonist and the immediate environment around the *P. gingivalis* determines whether Pg-LPS behaves as TLR4 evasive or suppressive [44]. In contrast, the ability of the LPS to activate TLR2 does not appear to depend upon the lipid A structure but instead is due to lipoproteins that are strongly associated with the hydrophilic polysaccharide fraction of the LPS [37]. The TLR2 activation of Pg-LPS (Wako) used in the present in vivo experiments was at the same levels as the Pg-LPS purchased from Invivogen and as in the Pg-LPS from *P. gingivalis* cultured with hemin, and at higher levels than *E. coli* LPS and Pg-LPS1435, suggesting that the Wako Pg-LPS has the ability to stimulate TLR2 signaling (Fig. 1). For the TLR4 activation, all the Pg-LPS tested here showed very much lower activities than *E. coli* LPS, suggesting that the ability of Wako Pg-LPS to stimulate TLR4 signaling is not nil but very low (Fig. 1). The Pg-LPS tested here is thought to be a TLR4 antagonist or to have little ability to stimulate and evade TLR4 sensing because it displays activity similar to antagonist Pg-LPS1435 for the TLR4-specific NF-kB reporter gene assay. Some lipoproteins may be co-purified with LPS due to their highly similar partitioning characteristics. Other lipoproteins may be covalently attached to the A-LPS fraction via the CTD sortase pathway and therefore contribute to the ability of PgLPS to act as a potent TLR2 agonist [45, 46].

### Influence of Pg-LPS on STZ-induced diabetic mice

All of the diabetic mice administered Pg-LPS were euthanized within the survival period of all of the non-diabetic mice administered Pg-LPS as did almost all of the diabetic mice not administered Pg-LPS, it may be considered that Pg-LPS raised the mortality of the diabetic mice (Fig. 2). The levels of urea nitrogen and creatinine in the blood, and urinary protein, were significantly higher in the diabetic mice administered Pg-LPS than in the diabetic mice not administered LPS at the humane endpoints of survival, suggesting that one of the causes of death may be nephropathy, and that pathogens of the periodontal disease *P. gingivalis* may cause nephropathy in diabetic patients (Fig. 2). There are reports that diabetic nephropathy has not occurred in TLR4 KO mice [30–35] but the TLR4 antagonist eritoran administration did not affect survival of diabetic mice administered Pg-LPS which has a poor ability to activate TLR4 signaling for Pg-LPS, suggesting that the Pg-LPS-induced diabetic nephropathy is dependent on both TLR2 and TLR4 signaling. Eritoran administration resulted in lower levels of urea nitrogen and creatinine in spite of the poor ability of Pg-LPS to activate TLR4. It has been fully elucidated that leukocytes and vascular endothelial cells are activated via TLR2 and TLR4 as nonspecific host defense systems [17–21]. It is thought that the repeatedly administered Pg-LPS activated circulating leukocytes via TLR2 and induced the production of inflammatory cytokines like interferon-γ. It may be suggested that intestinal gram-negative bacteria like *E. coli* were destroyed by the circulating activated leukocytes via TLR4 sensing, the membrane component LPS directly entering into the collateral circulation connecting enterohepatic circulation to the systemic circulation, and causing the progress of the nephropathy. TLR4 blocker eritoran may be able to slow down the progress of Pg-LPS-induced diabetic nephropathy by suppressing the activation of circulating leukocytes in glomeruli and glomerular endothelial cells via TLR4 sensing intestinal bacterial LPS.

Although eritoran administration resulted in lower levels of urea nitrogen and creatinine in the blood, and urinary protein of the surviving diabetic mice administered Pg-LPS, the increase in the survival rate of diabetic mice administered both Pg-LPS and eritoran was slight. There may be the possibility that the long-term stimulation of the immune system by Pg-LPS via TLR2 triggers inflammatory disorders in the lung or intestine to a higher extent than in diabetic mice not administered Pg-LPS. Eritoran could not protect diabetic mice administered LPS from deterioration in the general condition but eritroan may induce remission of nephropathy by protecting glomeruli from TLR4 ligands which are derived from the destroyed bacteria in the intestine or lung under the septic-like state caused by the long term immunization of Pg-LPS. There is also a possibility of chemical effects with eritoran acting on the glomeruli and further in vivo experiments using anti-TLR4 and eritoran are required.

### Expression of TLR2, TGF-β, and type 1 collagen in the diabetic mouse glomeruli

We have previously reported that vascular TLR2 expression is not present in non-diabetic mice or in non-diabetic mice administered Pg-LPS, but that it is observed in the diabetic mouse glomeruli [23]. Expression of pro-inflammatory cytokine interleukin (IL)-6 and tumor necrosis factor-α, as well as of anti-inflammatory cytokine TGF-β which promotes the type 1 collagen production, was detected only very weakly in non-diabetic mice whereas it was clearly detected in the diabetic mouse glomeruli and the amounts increased with Pg-LPS administration [22, 23]. In this study, there was little expression of TLR2 and TGF-β in the non-diabetic mice and in the non-diabetic mice administered Pg-LPS, but TLR2 and TGF-β were detected in the diabetic mouse glomeruli, and the expression was higher in the diabetic mice administered Pg-LPS (Figs. 3, 4, 6). Accumulation of type 1 collagen was higher in the diabetic mouse glomeruli than in the non-diabetic mice and in the non-diabetic mice administered Pg-LPS, and significantly higher in the diabetic mice administered Pg-LPS (Figs. 5, 6). These findings would suggest that Pg-LPS increases the TLR2 expression in glomeruli and activates glomerular host defense systems including leukocytes and endothelial cells via TLR2 sensing. The expressed levels of TLR2, TGF-β, and type 1 collagen were slightly lower in the diabetic mice administered both Pg-LPS and eritoran (Figs. 3, 4, 5, 6). The LPS from intestinal *E. coli*, which is destroyed by the circulating Pg-LPS-immunized leukocytes and entered into systemic circulation, may be recognized by glomerular capillary endothelial cells expressing TLR4 under diabetic conditions [17–21]. TLR4 blocker eritoran may be able to partially inhibit the activation of glomerular endothelial cells by the recognition of intestinal bacterial LPS via TLR4.

### Expression of STAT3 on the TLR2-positive glomeruli in diabetic mouse kidneys

The signal transducer and activator of transcription 3 (STAT3) is located in the cytoplasm in the non-activated state and in the Janus kinase (JAK)/STAT3 passage, STAT3 with tyrosine phosphorylation by activated JAK translocates to the nucleus, interacts with specific DNA elements, and functions as a transcription factor. Recently, STAT3-driven TLR2 and TLR4 up-regulation has been reported [47–50]. In the study here, STAT3 was not observed in the TLR2-negative glomeruli but both STAT3 and TLR2 were simultaneously detected in the diabetic mouse glomeruli (Fig. 7). The number of TLR2- and STAT3-positive glomeruli in the diabetic mice increased with Pg-LPS administration and double positive glomeruli numbers in the diabetic mice administered both Pg-LPS and eritoran were smaller than in the diabetic mice administrated Pg-LPS alone. The STAT3 was located in both cytoplasm and nucleus in the TLR2-positive cells, and TLR2 and STAT3 mRNAs were detected in the diabetic mouse renal cortex. The gene expression levels of TLR2 and STAT3 were higher with Pg-LPS administration but were lowered by the eritoran-administration (Fig. 8). These suggest that there was STAT3 accumulation in the cytoplasm and translocation to the nuclei in the diabetic mouse glomeruli and that this was promoted by Pg-LPS, and that the TLR2 expression in diabetic mouse glomeruli may be controlled by STAT3. Lower levels of TLR2 and STAT3 were observed in the diabetic mice administered both the TLR2 agonist Pg-LPS and TLR4 blocker eritoran than the levels of TLR2 and STAT3 in the diabetic mice administered Pg-LPS alone. In the host defense systems under the diabetic condition, the intestinal bacterial LPS may be produced by Pg-LPS-induced leukocyte immunization, enter into the systemic circulation, and stimulate glomerular TLR4 signaling-dependent STAT3 accumulation. Our results could be interpreted to suggest that STAT3-driven TLR2 production was partially suppressed by the eritoran-induced inhibition of TLR4-induced STAT3. In conclusion, the results would suggest that both TLR2 and TLR4 have the relevance to the promotion of *P. gingivalis*-induced nephropathy.
